# Characteristics of hip impingement syndrome in patients with multiple hereditary exostoses

**DOI:** 10.1186/s12891-021-04021-1

**Published:** 2021-02-06

**Authors:** Yeong-Seub Ahn, Sungmin Kim, Woo-Jong Kim, Jun-Hyuk Lim, Sung-Taek Jung

**Affiliations:** Department of Orthopaedic Surgery, Chonnam National University Hospital, Chonnam National University School of Medicine, 42 Jebongro, Donggu, Gwangju, 501-757 South Korea

**Keywords:** Impingement syndrome, Multiple hereditary exostoses, Coxa Valga, Acetabular dysplasia, Alpha angle, Minimum ischio-femoral distance

## Abstract

**Backgrounds:**

This study aimed to investigate the characteristic deformities of the hip in multiple hereditary exostoses patients (MHE) and its association with the hip impingement syndrome.

**Materials and methods:**

Between 2001 and 2019, total 51 patients (102 hips) were evaluated in this study. Patients with MHE were classified to femoro-acetabular impingement (FAI) symptom group, ischio-femoral impingement (IFI) symptom group and non-impingement symptom group by comparing the symptoms, clinical signs and imaging studies. To assess the morphometry of the hip in patients with MHE, the femoral neck-shaft angle, Sharp’s acetabular angle and center-edge (CE) angle were evaluated. Alpha angle was further evaluated to investigate the FAI using radiographs, and the minimum ischio-femoral distance was further measured to investigate the IFI using computed-tomographic (CT) study.

**Results:**

On hip impingement symptom analysis, FAI symptom and IFI symptom were confirmed in 14 hip joints and 18 hip joints, respectively. Unlike general population, the number of the hip with IFI-symptom was higher than those with FAI symptom in this study. In morphometric evaluation of MHE hips, coxa valga was most prominent deformity with occasional tendency of mild acetabular dysplasia. In a comparison of morphometric study between the impingement symptom group and non-symptom group, the FAI symptom showed significant differences of morphometric measure values than those of the non-symptom group (FAI symptom group vs. Non-FAI symptom group; Femoral neck-shaft angle (153.9 vs 142.6), Sharp’s angle (45.0 vs 41.5), CE angle (21.1 vs 28.8) and alpha angle (76.7 vs 57.9)). Similarly, the IFI symptom group also showed significant differences of morphometric measure values than those of the non-symptom group (IFI-symptom vs. Non-IFI symptom; Femoral neck-shaft angle (150.9 vs 142.7), Sharp’s angle (44.7 vs 41.4), CE angle (21.1 vs 29.3) and alpha angle (73.3 vs 56.8)). In addition, the minimum ischio-femoral distance measured using CT was significantly decreased in the IFI symptom group (IFI symptom group: 6.6, Non-IFI symptom group: 16.4).

**Conclusion:**

The results suggest that the characteristic deformities represented by coxa valga in the MHE hip act as an offset for FAI symptoms, on the contrary, act as a trigger for IFI symptoms.

**Level of evidence:**

Level III.

## Introduction

The hip impingement syndrome is generally classified into two categories. One, femoro-acetabular impingement (FAI), is already established as an intra-articular condition creating abnormal conflict between the acetabulum and femoral head-neck junction area. Over time, these kinds of frictions can damage the joint, causing pain and limiting activity [1]. The other one, ischiofemoral impingement (IFI), is defined as painful entrapment of the soft tissue between the ischium and femur lesser trochanter area [2–5]. In common, the symptoms of the two impingement syndromes are caused when two anatomical structures do not fit together perfectly or have insufficient distance to pass, continuously rubbing against each other during movement.

Currently, the concept of FAI with the prevalence being reported to range from 6 to 35% [6] is well established, and its treatment has considerably evolved [7–11]. However, studies on IFI syndrome are rarer than those on FAI syndrome. IFI as a clinical entity was first described in 1977 by Johnson [12], who reported on three patients with a complaint of pain after total hip replacement. Recently, the cause of IFI syndrome has been studied using various imaging techniques. Studies have reported that the dominant cause of IFI syndrome is the narrowing of the space between the ischial tuberosity and the lesser trochanter area, which can cause abnormal contact of the quadratus femoris muscle [2–5, 12, 13]. Although studies on IFI syndrome have been conducted recently, this syndrome has not been reported as much as FAI in general population.

On the basis of studies reporting that hip impingement syndrome is caused by an abnormal contact around the hip joint [1–5], this study postulated that the possible risk of developing hip impingement is higher in patients with multiple hereditary exostoses (MHE) exostoses, known to as developed at the metaphyseal area of bone due to endochondral ossification [14, 15]. Considering that the characteristic symptom of MHE is a palpable mass around the extremities causing limitation of joint motion, we thought that more studies of impingement syndrome in MHE patients would be reported in that MHE hip have a more susceptible environment to collisions caused by distinct lumps around the joints.

However, on evaluation of the previous studies of MHE impingement syndrome, there was no report of FAI in MHE patients, which is reported more in general population [6–11]. Rather, a few reports of IFI have been published [16, 17], which reported less than FAI in general population. Moreover, according to our findings, until recently, no studies have evaluated the association between hip deformities and hip impingement syndrome in patients with MHE.

This study, therefore, was conducted to investigate the hip impingement syndromes in MHE patients association with its characteristic hip deformities. Further, we tried to evaluate the correlation of the characteristic deformities with hip impingement syndrome in patients with MHE.

## Materials and methods

### Patients selection

This study retrospectively reviewed the patients diagnosed with MHE from January 1, 2001 to January 1, 2019. MHE patients with hip involvement confirmed by plain radiographs of the hip joints (those who had plain radiographs before surgical intervention or those who had not undergone any surgical intervention around the hip joints) were included. Patients with poor quality of radiographic images, those who underwent surgery elsewhere, and young patients without tri-radiate cartilage closure for the evaluation of mature hip joints were excluded.

### Impingement analysis

**Fig. 1:**
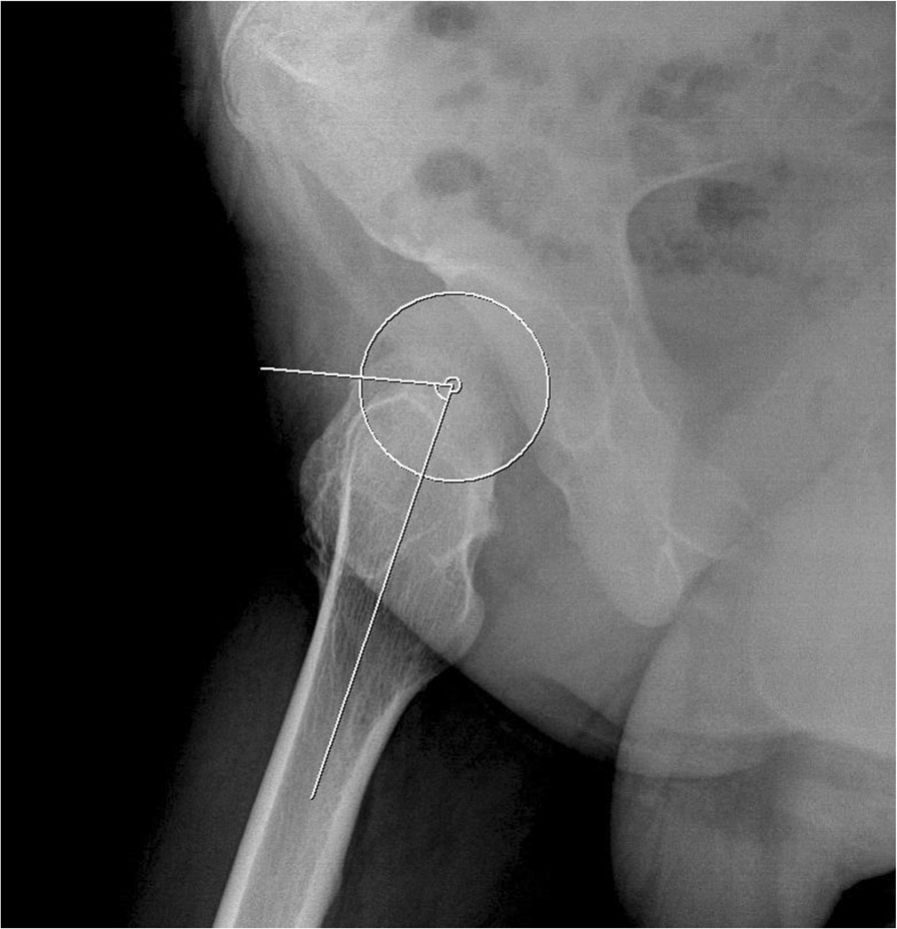
Measurement of the radiographic alpha angle. The alpha angle is determined by the angle between a line from the center of the femoral head through the middle of the femoral neck and a line through a point where the contour of the femoral head-neck junction exceeds the radius of the femoral head

Patients were classified into the FAI and IFI symptom groups by comparing the correlation with symptoms, physical exam and imaging studies. On FAI symptom patients evaluation, the patients who had a groin pain during the hip flexion, abduction and rotation, and had a corresponding images matched by alpha angle (Fig. 1) were defined as having FAI symptom. Whereas, the IFI symptom group defined as the patients who complained hip discomfort during hip extension, adduction, and rotation with a significant reduction of the minimum ischio-femoral distance on hip CT evaluation [18]. The patients who did not meet the FAI or IFI symptom were classified to non-symptom group.

### Morphometric analysis

**Fig. 2:**
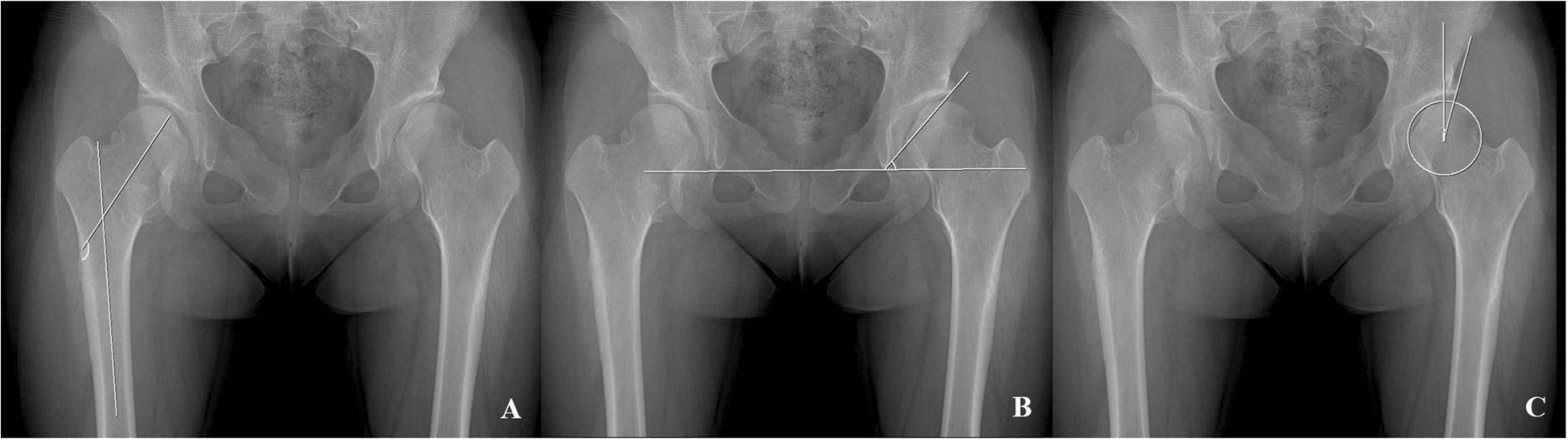
Plain radiographic measurements to evaluate the hip joint deformities. **a** The femoral neck-shaft angle is determined by measuring the angle created by a line in the central axis of femoral shaft and a second line created by the connection of the femoral head center to the mid portion of the femoral head and neck junction contour. **b** Sharp’s angle is determined by measuring the angle created by a line connecting the acetabular tear drops and a second line connecting a tear drop and the sourcil end. **c** The center-edge angle is determined by measuring the angle created by a line connecting the vertical line to the tear drop line through the center of the femoral head and a second line from the center of the hip to the lateral acetabular wall margin

**Fig. 3:**
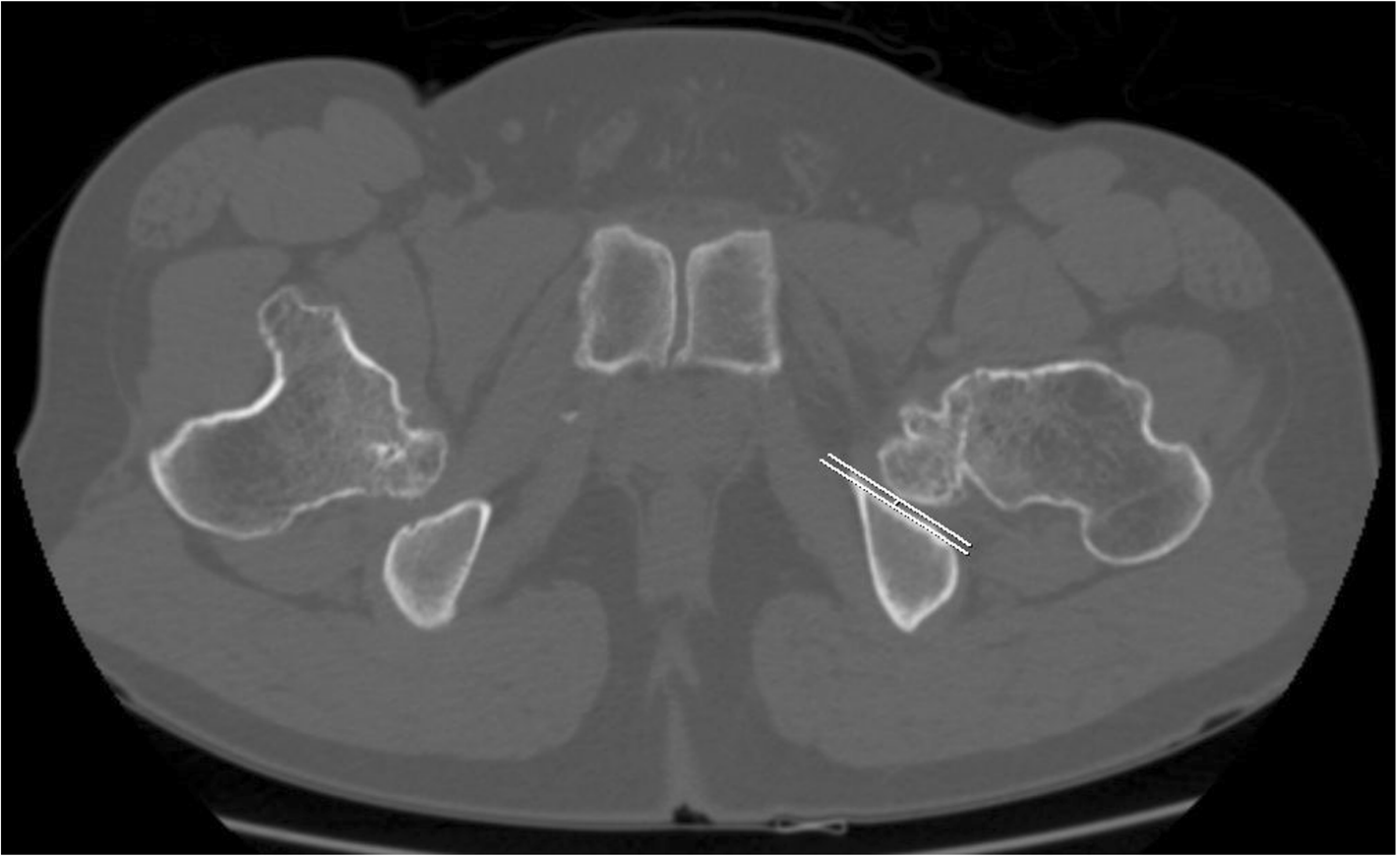
Measurement of minimum ischio-femoral distance to evaluate ischio-femoral impingement in a computed-tomographic study. The minimum ischio-femoral distance is determined by the nearest distance between the exostoses and ischium around the lesser trochanter area were measured at axial CT plane

Radiographic analysis was conducted using the last follow-up radiographs in patients who had not undergone any surgical intervention, and the last preoperative radiographs in those who had undergone surgical treatment around the hip joints. For the morphometry of the hip joint in patients with MHE, the femoral neck-shaft angle of Muller [19] was evaluated to assess proximal femur deformity, and Sharp’s acetabular angle [20] and the center-edge (CE) angle [21, 22] were evaluated to assess the deformities of acetabulum (Fig. 2). For FAI evaluation, alpha angle (Fig. 2) of all hips was measured regardless of the presence or absence of symptoms. For IFI evaluation, hip CT studies in patients with MHE were performed in the neutral supine position and the nearest distance between the exostoses and ischium around the lesser trochanter area were measured at axial plane (Fig. 3).

### Statistical analysis

Statistical analysis was performed using the SPSS version 21.0 software. The Mann-Whitney U-test was applied to compare the two differences according to the presence or absence of hip impingement symptoms. The correlation test was determined using spearman’s rank correlation method. Further, statistical significance was set at *P* <  0.05.

## Results

### Demographic data

**Fig. 4:**
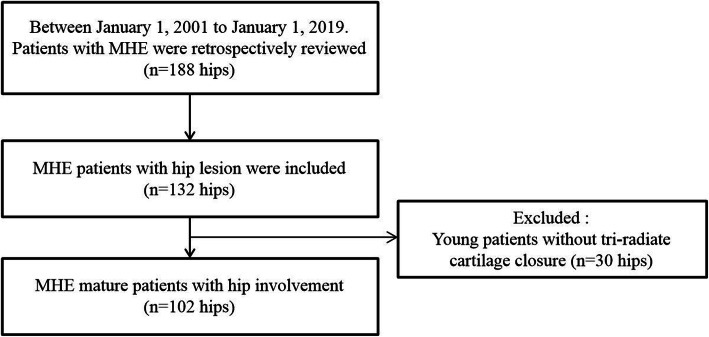
The figure shows how the MHE hip patients were recruited

Between 2001 and 2019, plain radiographs of 188 hips in 94 patients diagnosed with MHE were retrospectively reviewed. Among them, 28 patients (56 hips) who had no lesion around the hip joint were excluded. Although 132 hips in 66 patients (70.2%) were identified as having lesions around the hip joint, 15 young patients without tri-radiate cartilage closure were further excluded. Therefore, a total of 51 patients (102 hips) with tri-radiate cartilage closure were included (Fig. 4). The mean age of 51 patients (102 hips) was 24.0 (range, 14.0 to 54.0) years. Among them, 27 patients (54 hips) were men and 24 patients (48 hips) were women.

### Morphometric evaluation

In evaluation of the morphometric study of 102 MHE hips, the most common characteristic deformity was coxa valga with a mean femoral neck-shaft angle of 144.1° (range, 127.9° - 166.3°). The next remarkable features were that the sharp’s angle of 42.0° (range, 34.0° - 60.5°) on average was in the indeterminate range between the normal and acetabular dysplasia, and the CE angle, which means the femoral head coverage, was in the low normal range of 27.9° (range, 4.0° - 41.1°) on average.

### Morphometric study with FAI

On FAI evaluation, 62 MHE hips with an alpha angle of ≥55° which could be diagnosed as radiologically FAI [22] were observed. However, only 14 MHE hips were symptomatic during physical examination and were classified into FAI symptom group. As a result, the incidence of FAI symptom in MHE hips was confirmed to be 13.7% in this study. In comparison of morphometric study between the FAI-symptom and non FAI-symptom group, FAI symptom group showed more coxa valga deformity with a mean value of 153.9° (range, 143.8° - 161.1°) than that of non FAI-symptom group with a mean value of 142.6° (range, 127.9° - 166.3°) (*P* < .001), and showed more tendency of acetabular dysplasia presented by Sharp’s angle (FAI-symptom group: 45.0° (range, 31.5° - 61.5°); non FAI-symptom group: 41.5° (range, 34.0° - 53.6°); *P* = 0.027) and CE angle (FAI-symptom group: 21.1° (range, 4.0° - 41.1°); non FAI-symptom group: 28.8° (range, 9.3° - 38.8°); *P* = 0.017). In addition, the FAI symptom group showed an increased alpha angle (FAI-symptom group: 76.7° (range, 60.7° - 88.4°); non FAI-symptom group: 57.9° (range, 38.3° - 83.4°); *P* <  0.001) compared to that of the non FAI symptom group (Table 1).

**Table 1:** Comparison of morphometric study and body mass index between the femoro-acetabular (FAI) group and non-FAI group

Radiographic parameters	FAI group (*n* = 14)	Non-FAI group (*n* = 88)	*P* value
Femoral neck-shaft angle (°)	153.9 (143.8–161.1)	142.6 (127.9–166.3)	< 0.001
Sharp’s angle (°)	45.0 (31.5–60.5)	41.5 (34.0–53.6)	0.027
Center-edge angle (°)	21.1 (4.0–41.1)	28.8 (9.3–38.8)	0.017
Alpha angle (°)	76.7 (60.7–88.4)	57.9 (38.3–83.4)	< 0.001
Body mass index (kg/m2)	23.4 (16.7–27.5)	21.4 (16.0–35.3)	0.047

### Morphometric study with IFI

Eighteen of 102 MHE hips (17.7%) presented IFI symptom in this study. As a result of comparing and analyzing the morphometric studies of the IFI symptom and non-IFI symptom group, the IFI symptom group showed a similar deformity pattern compared to the study conducted between the FAI and non-FAI symptom groups. IFI symptom group showed the more coxa valga deformity (IFI-symptom group: 150.9° (range, 138.4° - 161.1°); non IFI-symptom group: 142.7° (range, 127.9° - 166.3°); *P* <  0.001), more tendency of acetabular dysplasia measured by Sharp’s angle (IFI-symptom group: 44.7° (range, 31.5° - 60.5°); non IFI-symptom group: 41.4° (range, 34.0° - 53.6°); *P* = 0.013) and CE angle (IFI-symptom group: 21.1° (range, 4.0° - 40.2°); non IFI-symptom group: 29.3° (range, 9.3° - 41.1°); *P* = 0.002), and more increased alpha angle (IFI-symptom group: 73.3° (range, 56.0° - 88.4°); non IFI-symptom group: 56.8° (range, 38.3° - 83.4°); P <  0.001) compared to that of the non IFI symptom group. Measured values to evaluate the deformities between the IFI and non IFI symptom group showed significant difference as noted in Table 2. For further study between the IFI and non IFI-symptom group, total 52 MHE hips being conducted hip CT including 18 MHE hips who had confirmed as having IFI symptom in physical examination were evaluated. In a further comparison between the IFI symptom and non IFI symptom group who underwent CT evaluation, the minimum ischio-femoral distance of the IFI-symptom group showed more decreased value with a mean value of 6.7 mm (range, 2.5–9.8) than that of non IFI-symptom group with a mean value of 16.4 mm (range, 10.2–25.2), and a significant difference (*P* < .001) was found (Table 3). In addition, minimum ischio-femoral distance was found to have a statistically significant reverse relations with the coxa valga deformity (*P* = 0.002), and as a result, it was confirmed that the minimum ischio-femoral distance decreased as the coxa valga deformity increased.

**Table 2:** Comparison of morphometric study and body mass index between the ischio-femoral impingement (IFI) group and non-IFI group

Radiographic parameters	IFI group (*n* = 18)	Non-IFI group (*n* = 84)	*P* value
Femoral neck-shaft angle (°)	150.9 (138.4–161.1)	142.7 (127.9–166.3)	< 0.001
Sharp’s angle (°)	44.7 (31.5–60.5)	41.4 (34.0–53.6)	0.013
Center-edge angle (°)	21.1 (4.0–40.2)	29.3 (9.3–41.1)	0.002
Alpha angle (°)	73.3 (56.0–88.4)	56.8 (38.3–83.4)	< 0.001
Body mass index (kg/m2)	22.2 (17.3–27.5)	21.6 (16.0–35.3)	0.347

**Table 3:** Comparison of morphometric study of the patients who had performed the computed-tomographic (CT) evaluation between the ischio-femoral impingement (IFI) group and non-IFI group

Radiographic parameters	IFI group (*n* = 18)	Non-IFI group (*n* = 34)	*P* value
Femoral neck-shaft angle (°)	151.5 (138.4–162.5)	144.6 (130.6–166.3)	0.008
Sharp’s angle (°)	44.7 (31.5–60.5)	40.7 (34.0–46.4)	0.013
Center-edge angle (°)	20.9 (4.0–40.2)	28.7 (9.3–41.1)	0.010
Alpha angle (°)	73.3 (56.0–88.4)	60.4 (38.3–83.4)	< 0.001
Minimum ischio femoral distance (mm)	6.7 (2.5–9.8)	16.4 (10.2–25.2)	< 0.001

## Discussion

As impingement syndrome in the hip joint can be caused by an abnormal contact between bony and soft tissue structures, this study postulated that the risk of hip impingement syndrome is higher in patients who have exostoses around the hip joints area. Generally, studies of FAI have been reported more than that of IFI in general patients who do not have MHE. Unlike the general populations, IFI have been reported more in patients with MHE [16, 17]. We deduced that the characteristic deformities of MHE hips with coexisting exostoses around the proximal femur may have a crucial role. Therefore, this study was conducted to investigate the deformities of the hip in patients with MHE and their relationship to hip impingement syndrome.

In general, the main characteristic deformities of MHE hips have been reported to be coxa valga in the proximal femur and occasional features of acetabular dysplasia [22]. Moreover, the presence of coxa valga has also been reported as a developmental consequence associated with acetabular dysplasia [21]. In present study, the main characteristic deformity of MHE hips was confirmed as coxa valga as similar to previous studies. In terms of the deformities in acetabular, acetabular dysplasia was not pathologically evident, however, it was found to belong to the upper normal range evaluated by sharp’s angle with occasional tendency of acetabular dysplasia [23].

**Fig. 5:**
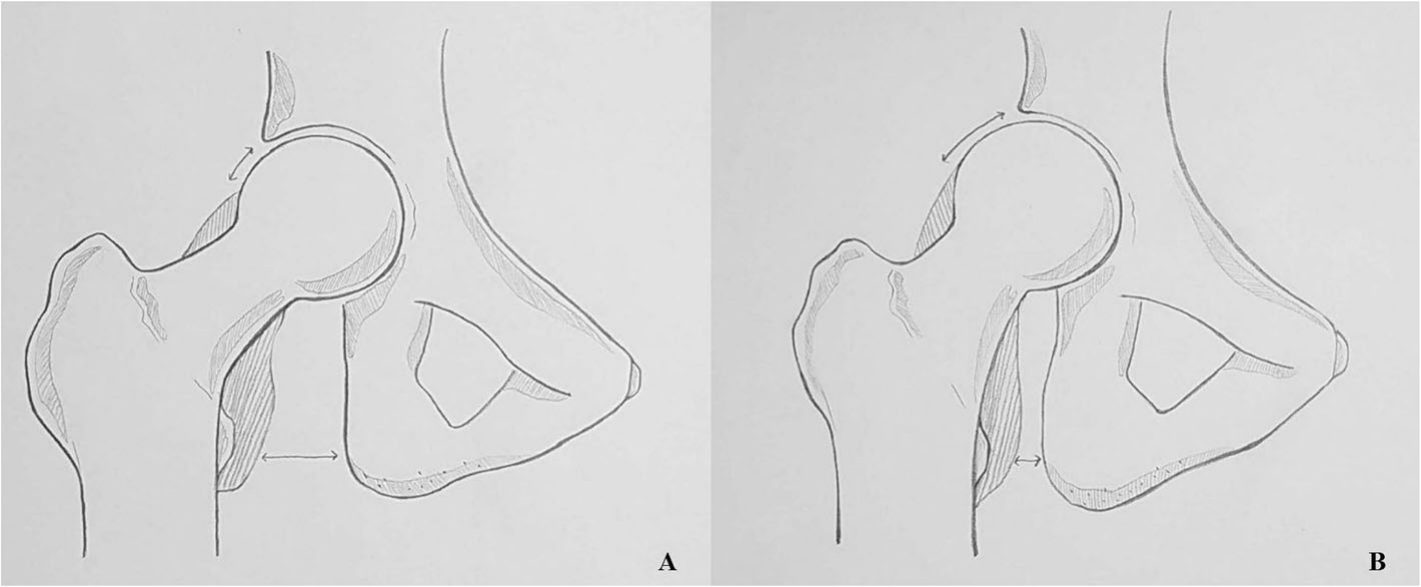
Illustration of the hip joint explaining why ischio-femoral impingement (IFI) symptoms are more common than femoro-acetabular (FAI) symptoms in hips with multiple hereditary (MHE). **a** The normal hip joint morphology without MHE. The hatched part in the figure shows the exostoses that can occur in the femoral head and neck area. In this circumstance, the incidence of both FAI and IFI is likely to increase. **b** MHE hip patients with the characteristic deformities. Though hatched part of the exostoses may increase the possibility of the impingement, coxa valga deformity reduces the possibility of impingement between the femoral head and acetabulum by increasing the working distance. On the contrary, coxa valga deformity acts as a risk factor for the development of IFI reducing the distance between the exostoses and the ischium

This study also investigated the relationship between impingement syndrome in MHE hips and the development of deformities. Considering the two measured values of Sharp’s angle and the CE angle in this study, the femoral head coverage by acetabular was confirmed to be reduced [24]. Therefore, pincer-type FAI caused by over-coverage of the femoral head seems less likely in patients with MHE. Instead, we postulated that the FAI in MHE patients was more likely to appear as a cam-type due to the exostoses around the femoral head-neck junction. According to a study on cam-type impingement syndrome in general patients, it was reported that the possibility of FAI is higher when the alpha-angle value is > 55° [25]. Although 62 MHE hips with an alpha angle of ≥55° were observed in this study, only 14 of 62 MHE hips presented symptomatic FAI. It is deduced that the reason only a small number of MHE hips exhibit FAI symptoms is because the increased alpha-angle values, which could play a role in conflicting with FAI, were compensated by other characteristic deformities of the MHE hips (Fig. 5). Therefore, it can be inferred that the characteristic of the MHE hip, which is represented by coxa valga deformity with tendency of acetabular dysplasia, increases the working distance between the proximal femur and acetabulum, which in turn plays a role of offset to the possibility of impingement.

Some studies [2–4, 24] have reported that IFI syndrome may result from the abnormal contact due to the reduced distance around the ischium and lesser trochanter area. Similarly, IFI in MHE hips has been reported to be caused by the shortening of the ischio-femoral distance due to exostoses around the lesser trochanter area and coxa valga deformities. In present study, the IFI symptom group showed a much higher incidence of coxa valga than the non IFI symptom group. In addition, referring to previous a study on MHE [24], Porter et al. reported that the occurrence of exostoses was more prevalent in the medial side of the proximal femur. As a result, the coxa valga deformity play a role as risk factor for IFI development encountered with the exostoses occurring around the lesser trochanter area. In the IFI symptom group in this study, the minimum ischio-femoral distance measured by hip CT was significantly decreased and coxa valga was significantly increased. Taken together, these characteristic deformities in MHE hips were thought to explain why more IFI symptoms appear in patients with MHE (Fig. 5).

There have been several limitation in this study. Though we thoroughly evaluated the symptoms of the patients complained during physical examination, more precise diagnostic tools such as MR arthrography or joint injection had not performed to rule out other possible pathology around hip joint in this study. In the evaluation of the patients with IFI symptom, this study have limitation in that CT studies were not performed in all asymptomatic patients. In addition, there may be limitations in distinguishing soft tissue impingement or bony impingement owing to the use of CT rather than MRI. In clinical aspect, studies on the degree of deformity and pain in patients are lacking, so further studies will be considered to be additionally needed.

However, this study is meaningful in that it investigated the association between the characteristic deformities of MHE hip joints and impingement syndrome. Furthermore, this study also showed that the most characteristic deformity in MHE hips is coxa valga regardless of the impingement type, often accompanied by mild acetabular dysplasia. Further, this study confirmed that the characteristics of the deformities act as differently in each type of impingement syndrome.

## Conclusions

MHE patients were thought to be more likely to develop impingement symptoms due to the exostoses. However, IFI symptoms, which are uncommon in the general populations, were more common in MHE patients, and FAI reported a relatively smaller number than IFI. This study confirmed that the characteristic deformities represented by coxa valga in MHE hips act as an offset to FAI symptom, conversely, trigger to IFI symptom.

## Data Availability

The datasets used and/or analyzed during the current study are available from the corresponding author on reasonable request.

## Abbreviations

MHE: Multiple hereditary exostoses
CE: Center-edge
FAI: Femoro-acetabular impingement
IFI: Ischio-femoral impingement
CT: Computed-tomography
